# Epidemiology, Risk Factors, and Prophylaxis Use for *Pneumocystis jirovecii* Pneumonia in the Non-HIV Population: A Retrospective Study in Québec, Canada

**DOI:** 10.1093/ofid/ofad639

**Published:** 2023-12-18

**Authors:** Nicholas Quigley, Laurence d’Amours, Philippe Gervais, Geneviève Dion

**Affiliations:** Department of Pulmonary Medicine, Institut Universitaire de Cardiologie et de Pneumologie de Québec (Québec Heart and Lung Institute), Université Laval, Québec City, Québec; Department of Critical Care Medicine, Faculty of Medicine and Dentistry, University of Alberta, Edmonton, Alberta; Department of Pulmonary Medicine, Institut Universitaire de Cardiologie et de Pneumologie de Québec (Québec Heart and Lung Institute), Université Laval, Québec City, Québec; Department of Microbiology and Infectious Diseases, Institut Universitaire de Cardiologie et de Pneumologie de Québec (Québec Heart and Lung Institute), Université Laval, Québec City, Québec, Canada; Department of Pulmonary Medicine, Institut Universitaire de Cardiologie et de Pneumologie de Québec (Québec Heart and Lung Institute), Université Laval, Québec City, Québec

**Keywords:** corticosteroids, immunocompromised, *Pneumocystis jirovecii*, pneumonia, prophylaxis

## Abstract

**Background:**

*Pneumocystis jirovecii* pneumonia (PJP) remains a significant threat in immunocompromised cases. Recent data on epidemiology and risk factors for PJP in non-HIV cases are scarce, and guidelines on appropriate prophylaxis are lacking.

**Methods:**

In this multicenter retrospective trial, all non-HIV adult cases admitted to hospitals in Québec City, Canada, between January 2011 and January 2021 with a diagnosis of PJP were assessed for eligibility.

**Results:**

An overall 129 cases of PJP were included. More than two-thirds had an underlying hematologic disease or an autoimmune/inflammatory condition. Prior to diagnosis, 83.7% were taking corticosteroids, 71.3% immunosuppressive agents (alone or in combination with corticosteroids), and 62% both. A diagnosis of PJP was noted in 22 patients receiving corticosteroids for treatment <28 days. Two patients developed PJP while undergoing corticosteroid monotherapy at a mean daily prednisone-equivalent dose <20 mg/d; 4.7% of our cohort received a PJP prophylaxis. Current recommendations or accepted clinical practices for PJP prophylaxis would not have applied to 48.8% of our patients.

**Conclusions:**

The use of corticosteroids—in monotherapy or in coadministration with other immunosuppressive agents—remains the principal risk factor for PJP in the non-HIV population. Current prophylaxis guidelines and accepted practices are insufficient to adequately prevent PJP and need to be broadened and updated.

## Teaser

Corticosteroids—in monotherapy or in coadministration with other immunosuppressive agents—are the principal risk factor for *Pneumocystis jirovecii* pneumonia in the non-HIV population. Current prophylaxis guidelines and accepted practices are insufficient to adequately prevent *P jirovecii* pneumonia and need to be updated.

## BACKGROUND

The 1980s saw a fulgurant rise in the number of *Pneumocystis jirovecii* pneumonia (PJP) cases along with the HIV epidemic [1]. Since then, multiple studies and practice guides have defined the most appropriate approach to diagnosis, treatment, and prevention of this opportunistic fungal infection in this specific population [2]. The advent of effective antiretroviral therapies combined with the general adoption of recommendations for *Pneumocystis* prophylaxis in patients with HIV has led to reduced rates of PJP in this population. Notwithstanding this change in epidemiology [3], *P jirovecii* remains a significant cause of pneumonia in patients with other types of immunodeficiencies. An increasing number of individuals are now treated with immunosuppressive drugs and corticosteroids for inflammatory or autoimmune conditions [4, 5]. Furthermore, newer immunosuppressive drugs are now marketed, and the number of drugs associated with an increased risk for PJP is constantly expanding. It is therefore no wonder that cases of PJP in the non-HIV population are growing [6–8].

Despite improvements over the last 4 decades in the treatment of PJP, it remains a serious infection with often dramatic consequences. The disease can be particularly severe in non-HIV cases, for which hypoxemia and lung inflammation are more prominent [9] and mortality is twice that of HIV infection at 30% to 60% [10, 11].

While guidelines exist about effective PJP prophylaxis for patients with cancer and recipients of solid organ or hematopoietic cell transplant [12, 13], non-HIV cases of PJP have not been as extensively studied, especially in recent years [3], and uncertainties remain regarding an optimal approach for other patients who are immunocompromised. Although PJP risk has been correlated with the level of immunosuppression [7], there are still uncertainties about the specific agents or the corticosteroid cutoff dose or duration that justify the use of a prophylaxis [8].

Considering the relative paucity of recent literature on the epidemiology and risk factors for PJP in non-HIV cases, we conducted a retrospective study in a modern North American setting. Our principal objective was to provide clinicians with up-to-date data on the associated pathologies and level of immunosuppression of PJP cases that may ultimately influence the use of prophylaxis. The clinical evolution and management of PJP in our cohort of patients who were HIV negative were also described.

## METHODS

We performed a retrospective observational study of all patients admitted with a diagnosis of PJP to the 6 teaching hospitals of Québec City (Québec, Canada) between January 2011 and January 2021. These centers offer tertiary care to the population of eastern Québec with a catchment area of approximately 2 million people. Affected patients were identified by a computerized search of the medical records at each medical center. Approval was obtained from the institutional research ethics boards of the Institut Universitaire de Cardiologie et de Pneumologie de Québec and the Centre Hospitalier Universitaire de Québec.

PJP diagnosis required (1) compatible clinical symptoms and (2) confirmatory microbiological testing defined by positive immunofluorescence staining on bronchoalveolar lavage or tissue samples or quantitative polymerase chain reaction (PCR) assay on respiratory specimens. PCR-positive cases were all reviewed and discussed among authors to minimize the possibility of including patients with *P jirovecii* colonization. Presumptive cases without microbiological proof were excluded as well as patients <18 years of age and those with HIV.

Population baseline characteristics were collected with specific features related to PJP. Details were explicitly sought on associated conditions, immunosuppressive treatment, use of corticosteroids, and prescription of a PJP prophylaxis. Studied outcomes were the duration of hospital stay, intensive care unit (ICU) admission, the use of mechanical ventilation, and in-hospital mortality. We used descriptive statistics and performed univariate and multivariate analysis.

## RESULTS

**Figure 1.**
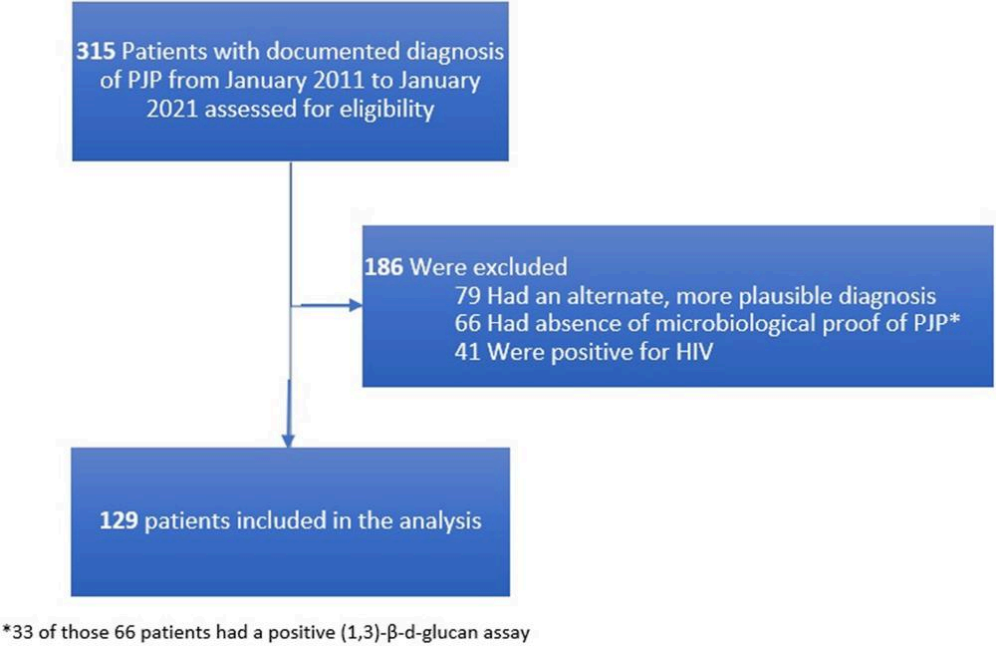
Flowchart of screening and inclusion of patients. *Of those 66 patients, 33 had a positive (1,3)-β-d-glucan assay. PJP, *Pneumocystis jirovecii* pneumonia.

An overall 315 potential cases were initially screened for the study period of January 2011 to January 2021 (Figure 1). Forty-one patients proved to be HIV positive, and 79 with a more plausible alternate diagnosis were excluded. Sixty-six additional cases were excluded in the absence of prespecified microbiological proof of PJP. Among them, 33 had a positive (1,3)-β-d-glucan assay result. A total of 129 cases were included in the analysis. The final diagnosis was supported by immunofluorescence from a bronchoalveolar lavage in 95 patients (73.6%). *P jirovecii* was identified by PCR assay in 33 patients (25.6%), and a single case (0.8%) had its diagnosis on autopsy. Baseline characteristics of the studied patients are summarized in Table 1.

**Table 1.** Baseline Patient Characteristics, Underlying Conditions, and Therapies

Characteristic	No. (%)
Patients	129
Female	53 (41.1)
Age, y, mean (SD)	66.5 (14.1)
Smoking	
Active	6 (4.7)
Previous	60 (46.5)
Pack-years, mean (SD)	31.3 (18.7)
Diabetes	31 (24.0)
Chronic obstructive pulmonary disease	15 (11.6)
Body mass index, mean (SD)	24.9 (4.3)
Underlying conditions	
Hematologic diseases	55 (42.6)
Lymphoma	32
Hematopoietic stem cell transplant	9
Multiple myeloma	7
Leukemia	
Acute	4
Chronic lymphocytic	1
Chronic myelomonocytic	1
Hemophagocytic lymphohistiocytosis	1
Autoimmune/inflammatory diseases	37 (28.7)
Rheumatoid arthritis	12
Interstitial lung disease	10
Renal inflammatory disease	3
Dermatomyositis	2
Psoriatic arthritis	2
Other autoimmune/inflammatory diseases	8
Solid tumors	17 (13.2)
Lung	12
Breast	2
Central nervous system	2
Kidney	1
Solid organ transplants	15 (11.6)
Kidney	11
Heart	3
Combined heart-kidney	1
Other^a^	3 (2.3)
None identified	2 (1.6)
Underlying therapies	
Corticosteroid use	108 (83.7)
Immunosuppressive agent use	
Excluding corticosteroid	92 (71.3)
With corticosteroid	80 (62.0)
No immunosuppressive agent or corticosteroid	9 (7.0)
PJP prophylaxis use	6 (4.7)

Data are presented as No. (%) unless noted otherwise.

Abbreviation: PJP, *Pneumocystis jirovecii* pneumonia.

^a^All 3 patients received corticosteroid therapy in the setting of craniectomy following a subarachnoid hemorrhage (n = 1), intraparenchymal hemorrhage with suspected vasogenic edema (n = 1), and symptomatic discal hernia (n = 1).

Hematologic diseases were the most frequently associated underlying condition (42.6%), followed by autoimmune and inflammatory processes (28.7%), solid malignancies (13.2%), and solid organ transplants (11.6%). Lymphoma was the most frequently associated underlying condition, accounting for 24.8% (32/129) of all cases. When lymphoma was added to rheumatoid arthritis (n = 12), lung cancer (n = 12), kidney transplants (n = 11), interstitial lung diseases (n = 10), and hematopoietic stem cell transplants (n = 9), they together represented two-thirds (86/129) of cases of PJP. In sum, 108 patients (83.7%) received corticosteroids. Among them, 28 (25.9%) were undergoing corticosteroid therapy alone, and 80 (74.1%) were receiving another immunosuppressant agent. Ninety-two patients (71.3%) were undergoing immunosuppressive drug therapy alone or in combination with corticosteroids prior to the diagnosis of PJP. Among them, 80 (87.0%) also received corticosteroid therapy, and 47 (51.1%) were taking >1 immunosuppressive agent (excluding corticosteroids). Nine patients (7.0%) had no recent corticosteroid or other immunosuppressive exposure. Of them, 7 had underlying conditions classically associated with PJP (Supplementary Table 1). For the other 2 patients, no underlying risk factor or associated condition could be identified. Of note, 14 of the 15 transplant recipients who developed PJP were diagnosed more than a year after their transplantation.

Among the 108 patients who received corticosteroids prior to being diagnosed with PJP, the median duration of corticosteroid therapy was 71.5 days, and the mean prednisone-equivalent dose was 34.7 mg/d (Table 2). Those undergoing corticosteroid monotherapy had a higher mean prednisone-equivalent dose than those who were taking another immunosuppressive agent (40.6 vs 32.6 mg/d). Their median duration of corticosteroid therapy prior to diagnosis was shorter at 40.0 days vs 92.5 days for those prescribed combined therapy. Among the 22 individuals who received corticosteroids for <28 days (at a mean daily prednisone-equivalent dose of 53.2 mg), 10 did not receive other immunosuppressive therapy, and 2 had corticosteroid therapy for <14 days. For the 10 patients not receiving other immunosuppressive agents, the mean daily prednisone-equivalent dose was 34.4 mg. For the 2 patients receiving corticosteroid therapy for <14 days, the mean daily dose was 57.7 mg. Of note, 30 of the 80 patients (37.5%) undergoing combined corticosteroid and another immunosuppressive therapy were taking mean prednisone-equivalent doses <20 mg/d, with 17 receiving <10 mg/d and 13 between 10 and 20 mg/d. Only 2 patients developed PJP while taking corticosteroids alone at a mean daily prednisone-equivalent dose <20 mg (15.3 and 17.3 mg/d).

**Table 2.** Corticosteroid and Immunosuppressive Use in Patients With PJP

	Immunosuppressive Agent		
	No	Yes	Overall
No CS use	9	12	21
CS use	28	80	108
CS therapy, d, median (IQR)	40.0 (22.3–55.3)	92.5 (47.8–316.3)	71.5 (34.5–133)
Daily CS dose, mg, mean (SD)^a^	40.6 (17.1)	32.6 (30.6)	34.7 (27.9)
CS therapy <28 d	10 (35.7)	12 (15.0)	22 (20.4)
Mean daily CS dose <20 mg^a^	2 (7.1)	30 (37.5)	32 (29.6)
CS therapy <28 d and mean daily CS dose <20 mg^a^	2 (7.1)	1 (1.3)	3 (2.8)

Data are presented as No. (%) unless noted otherwise.

Abbreviations: CS, corticosteroid; PJP, *Pneumocystis jirovecii* pneumonia.

^a^Prednisone equivalent.

Immunosuppressive agents other than corticosteroids were used in all solid organ transplant recipients with PJP and 83.6% of patients with hematologic diseases. They were less commonly used in patients whose underlying conditions were autoimmune or inflammatory diseases (59.5%) and solid tumors (52.9%). Details on the immunosuppressive agents used are outlined in Table 3. Among patients with hematologic diseases receiving immunosuppressive agents, either a combination of monoclonal antibody and cytotoxic chemotherapy or cytotoxic chemotherapy alone was the most encountered, accounting for 73.9% (34/46) of cases. Methotrexate, in mono- or combination therapy, was the most common immunosuppressive agent in patients with autoimmune or inflammatory conditions. In patients with solid tumors, cytotoxic chemotherapy was most frequent. The use of a calcineurin inhibitor with an antiproliferative agent was noted in 60.0% (9/15) of solid organ transplant recipients with PJP. Coadministration of corticosteroids with immunosuppressive agents was noted in 93.3% of solid organ transplant recipients, 74.5% of patients with hematologic conditions, and 47.1% and 45.9% of those with solid tumors and autoimmune or inflammatory diseases, respectively. Only 6 patients (4.7%), all with an underlying hematologic disease, were undergoing PJP prophylaxis at the time of diagnosis. Their characteristics are detailed in Supplementary Table 2.

**Table 3.** Details on Use of Immunosuppressives in Patient With PJP According to Underlying Conditions

	Hematologic Diseases	Autoimmune/Inflammatory Diseases	Solid Tumors	Solid Organ Transplants	Other	Overall
PJP cases according to underlying conditions	55	37	17	15	5	129
Immunosuppressive agent use^a^	46 (83.6)	22 (59.5)	9 (52.9)	15 (100)	0	92 (71.3)
Immunosuppressive agent (coadministration of steroids)^b^	Monoclonal antibody + cytotoxic chemotherapy: 21 (19) Cytotoxic chemotherapy: 13 (12) Calcineurin inhibitor: 4 (2) Tyrosine kinase inhibitor: 2 (2) Monoclonal antibody: 2 (2) Protease inhibitor + cytotoxic chemotherapy: 2 (2) Others: 2 (2)	MTX: 7 (4) MTX + HCQ: 3 (3) MTX + monoclonal antibody: 3 (2) Antiproliferative agent: 1 (1) HCQ + LEF + SSZ: 1 (1) HCQ: 1 (1) LEF: 1 (1) Monoclonal antibody: 1 (1) Other combinations including HCQ and/or MTX: 4 (3)	Cytotoxic chemotherapy: 6 (6) Tyrosine kinase inhibitor: 2 (2) Others: 1 (0)	Calcineurin inhibitor + antiproliferative agent: 9 (9) Calcineurin inhibitor + mTOR inhibitor: 1 (1) Calcineurin inhibitor: 1 (0) Antiproliferative agent: 1 (1) Other combinations including a calcineurin inhibitor: 3 (3)		
Immunosuppressive agents and coadministration of corticosteroids	41 (74.5)	17 (45.9)	8 (47.1)	14 (93.3)		80 (62)
PJP prophylaxis used	6 (10.9)	0	0	0	0	6 (4.7)

Antiproliferative agents include mycophenolic acid and azathioprine. Data are presented as No. (%) unless noted otherwise.

Abbreviations: HCQ, hydroxychloroquine; LEF, leflunomide; mTOR, mammalian target of rapamycin; MTX, methotrexate; PJP, *Pneumocystis jirovecii* pneumonia; SSZ, sulfasalazine.

^a^Exclusive of corticosteroids.

^b^The first number refers to the number of patients taking the immunosuppressive agent exclusive of corticosteroids; the second number refers to the number of patients being coadministered steroids.

Forty-two individuals treated with immunosuppressive agents and diagnosed with PJP were receiving a mean prednisone-equivalent dose <20 mg/d (n = 30) or no corticosteroids (n = 12) (Table 4). Among the 12 patients who developed PJP while taking immunosuppressive agents alone (ie, without the coadministration of steroids), 5 had hematologic conditions and 5 had autoimmune or inflammatory diseases (Supplementary Table 3).

**Table 4.** Immunosuppression of Patients With PJP Receiving Either No Corticosteroid or Corticosteroid Therapy <20 mg Daily

	No. of Patients	
Associated Condition: Comedication	Corticosteroid Therapy <20 mg Daily	Not Receiving Corticosteroids
Hematologic disease	10	9
Monoclonal antibody + cytotoxic chemotherapy	4	2
Cytotoxic chemotherapy	2	1
Calcineurin inhibitor	0	2
Protease inhibitor + cytotoxic chemotherapy	1	0
Other	2	0
No immunosuppressive agent	1	4
Autoimmune/inflammatory disease	7	7
MTX	1	3
MTX + monoclonal antibody	2	1
HCQ + LEF + SSZ	1	0
HCQ	1	0
LEF	1	0
Other combinations including HCQ and/or MTX	1	1
No immunosuppressive agent	0	2
Solid tumors	2	2
Cytotoxic chemotherapy	1	0
No immunosuppressive agent	1	1
Other	0	1
Solid organ transplants	13	1
Calcineurin inhibitor + antiproliferative agent	9	0
Calcineurin inhibitor + mTOR inhibitor	1	0
Calcineurin inhibitor	0	1
Antiproliferative agent	1	0
Other combinations including a calcineurin inhibitor	2	0
Others	0	0
No underlying conditions identified	0	2
No immunosuppressive agent	0	2
Total	32	21

Corticosteroid therapy is based on a prednisone-equivalent dose.

Abbreviations: HCQ, hydroxychloroquine; LEF, leflunomide; mTOR, mammalian target of rapamycin; MTX, methotrexate; PJP, *Pneumocystis jirovecii* pneumonia; SSZ, sulfasalazine.

An overall 110 patients (85.2%) were treated with trimethoprim-sulfamethoxazole (TMP-SMX) for their infection, while 14 (10.9%) received a combination of primaquine and clindamycin as first-line therapy (Table 5). Forty-two patients (38.2%) receiving TMP-SMX required a switch to other therapies due to side effects. Furthermore, 110 patients (85.2%) received corticosteroids as an adjunctive therapy [14]. The mean number of days in hospital was 19.5. In addition, 39.5% of patients (51/129) were admitted to the ICU, where the mean length of stay was 9.0 days. Invasive mechanical ventilation was required in 24 patients (18.6%) for a mean duration of 8.4 days. In-hospital mortality was 31.0%. Patients with underlying solid tumors were more likely to die after PJP diagnosis (70.6% in-hospital mortality). In the subgroup of patients admitted to the ICU, in-hospital mortality rose to 49% (25/51), though it was even higher in those requiring mechanical ventilation at 58.3% (14/24).

**Table 5.** PJP Treatment and Outcomes

	No. (%)
Pharmacologic therapies	
First-line treatment	
TMP-SMX	110/129 (85.2)
Primaquine + clindamycin	14/129 (10.9)
Atovaquone	2/129 (1.55)
Pentamidine	1/129 (0.78)
No antimicrobial/palliative care	2/129 (1.55)
Adverse events under TMP-SMX prompting a change to other antimicrobials	42/110 (38.2)
Corticosteroids as initial adjunctive therapy	110/129 (85.2)
Outcomes	
Days in hospital, mean (SD)	19.5 (17.1)
ICU admission	51/129 (39.5)
Days in ICU, mean (SD)	9.0 (6.9)
Invasive mechanical ventilation	24/129 (18.6)
Days of invasive mechanical ventilation, mean (SD)	8.4 (6.7)
In-hospital mortality	40/129 (31.0)
Hematologic diseases	11/55 (20.0)
Autoimmune/inflammatory diseases	12/37 (32.4)
Solid tumors	12/17 (70.6)
Solid organ transplants	3/15 (20.0)
Others	1/3 (33.3)
Unidentified	1/2 (50.0)
Admitted to ICU	25/51 (49.0)
Invasive mechanical ventilation	14/24 (58.3)

Data are presented as No. (%) unless noted otherwise.

Abbreviations: ICU, intensive care unit; PJP, *Pneumocystis jirovecii* pneumonia; TMP-SMX, trimethoprim-sulfamethoxazole.

## DISCUSSION

Our descriptive retrospective study examined non-HIV cases of PJP over 10 years. More than two-thirds of our patient population had an underlying hematologic disease or an autoimmune/inflammatory condition, which is consistent with the most recent reports of non-HIV cases published in the literature by Calero-Bernal et al [8] and Nunes et al [15]. The in-hospital mortality rate of 31% emphasizes the gravity of the condition and is in concordance with the reported 30%–60% mortality over the last 2 decades [10, 11].

Corticosteroids and immunosuppressive therapy have long been recognized as major risk factors for the development of PJP, with corticosteroids use preceding PJP diagnosis independently associated with increased mortality [16]. Unsurprisingly, as previously reported [8, 15], a majority (93%) of patients were undergoing medical therapy targeting their immune system, with corticosteroids being more commonly prescribed than other immunosuppressive drugs. Sixty-two percent (80/129) of our patient population had a combination of immunosuppressive and corticosteroid therapy. The use of PJP prophylaxis was minimal (4.7% of patients) and comparable to prior reports by Calero-Bernal (5.5%) [8] and Nunes et al (5%) [15].

Only a few medical societies have published clinical guidelines on the use of prophylaxis to prevent PJP in the non-HIV population. The American Society of Transplantation recommends PJP prophylaxis for at least 6 to 12 months posttransplant and lifelong prophylaxis for lung and small bowel transplant recipients [17]. The Fifth European Conference on Infections in Leukemia recommended in 2016 that patients should receive PJP prophylaxis if they (1) have acute lymphoblastic leukemia or allogeneic hematopoietic stem cell transplantation; (2) are being treated with alemtuzumab or combinations of fludarabine, cyclophosphamide, and rituximab; or (3) are receiving corticosteroids at prednisone-equivalent doses of 20 mg daily for >4 weeks [18]. According to the German Society of Hematology and Medical Oncology, special considerations for prophylaxis are also given to selected patients with autologous hematopoietic stem cell transplantation, patients treated with nucleoside analogs or idelalisib, and those receiving brain irradiation with temozolomide or high-dose corticosteroids [19]. The American Society of Clinical Oncology and the Infectious Diseases Society of America recommend PJP prophylaxis for patients receiving chemotherapy regimens that are associated with a >3.5% risk of developing PJP [20] based on results from an older meta-analysis by Green et al [21]. However, with continuously evolving chemotherapy regimens, data on the associated PJP risk are often not easily accessible to clinicians. Although individuals with autoimmune or inflammatory diseases account for a significant proportion of PJP cases in our patient population (28.7%), there is currently no formal recommendation for prophylaxis for them. Regardless, we are witnessing growing acceptance for the use of PJP prophylaxis among such patients when receiving high-dose corticosteroids, especially in conjunction with other immunosuppressive agents [22–24].

**Figure 2.**
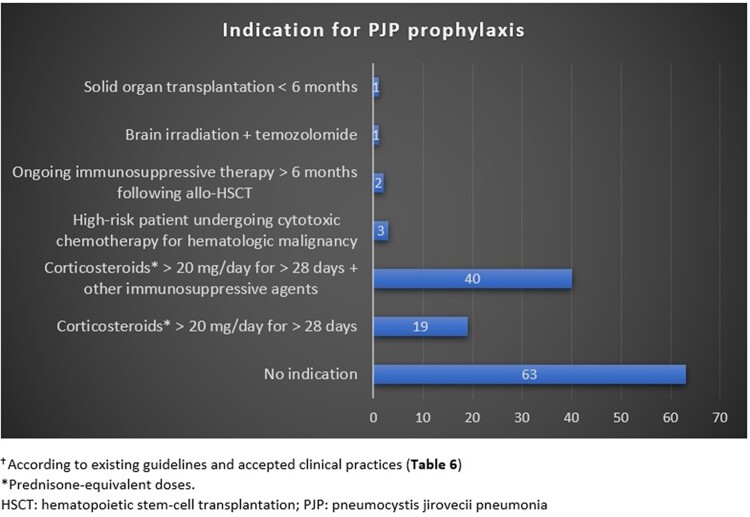
Patients for whom PJP prophylaxis would have been indicated according to existing guidelines and accepted clinical practices (Table 6). *Prednisone-equivalent doses. HSCT, hematopoietic stem cell transplantation; PJP, *Pneumocystis jirovecii* pneumonia.

We reviewed all cases of our cohort in light of those guidelines and accepted practices [17–24] (Table 6) and assessed whether patients would have qualified for PJP prophylaxis based on them. We found that 66 of our 129 patients (51.2%) met actual criteria or accepted clinical practices for prophylaxis (Figure 2), although only 6 (9.1%) received it. Regardless of the underlying conditions, the use of corticosteroids at prednisone-equivalent doses >20 mg/d for >28 days alone or in association with other immunosuppressive agents accounted for the majority of indications or accepted clinical practices. Remarkably, current recommendations or accepted practices for PJP prophylaxis would not have applied to 48.8% of our patients. Among them, it is intriguing to notice that patients prescribed multiple immunosuppressive agents and those taking immunosuppressive agents in combination with corticosteroids at <20 mg/d or for <28 days would not even be considered despite representing 36.4% (47/129) and 31.8% (41/129) of our PJP cases, respectively. Interestingly, 12 patients (9.1%) in our cohort and 13% in the Calero-Bernal et al cohort [8] developed PJP while taking immunosuppressive agents without coadministration of corticosteroids. These findings strongly suggest that while corticosteroid therapy is a strong risk factor for the development of PJP, patients treated only by immunosuppressive agents, without steroids, also present a risk of PJP. Furthermore, the use of corticosteroids, regardless of the dose, appears to be associated with an increased risk of PJP, when taken in association with another immunosuppressive agent. Indeed, in our cohort, 30 patients (37.5%) developed PJP while receiving immunosuppressive agents combined with corticosteroids at a mean prednisone-equivalent dose <20 mg/d: 17 patients received corticosteroids at doses varying from 10 to 20 mg/d and 13 patients at doses inferior to 10 mg/d in combination with immunosuppressive therapy. This finding suggests that there is no safe dose of corticosteroids when taken in combination with another immunosuppressive agent. Regarding patients treated only with corticosteroids, while doses and duration of corticosteroid therapy varied significantly, 92.9% (26/28) received corticosteroids at a dose superior to or equal to 20 mg/d of prednisone. Although 2 patients developed PJP during corticosteroid monotherapy at a dose <20 mg/d, none had a daily prednisone-equivalent dose inferior to 15 mg. This is in accordance with the study from Yale and Limper [3], who reported that a daily dose of 16 mg over a median 8 weeks increased the risk of developing PJP, suggesting a lower threshold for increased risk of PJP. Moreover, in our cohort, 10 patients (7.7%) were treated with corticosteroids alone for <28 days, which is higher than the 3 of 128 cases (2.3%) reported by Calero-Bernal et al [8]. Only 2 patients developed PJP following corticosteroid monotherapy for <14 days, which could represent a more conservative cutoff for corticosteroid treatment duration leading to a higher risk of PJP.

**Table 6.** Current Indications and Accepted Clinical Practices for PJP Prophylaxis for Non-HIV Immunocompromised Cases

Cases	Indication for PJP Prophylaxis
Hematologic malignancies^a^	Corticosteroids at prednisone-equivalent doses >20 mg/d for >4 wk Acute lymphoblastic leukemia with chemotherapy T-cell–depleting agents or fludarabine/cyclophosphamide/rituximab combinations Alemtuzumab Idelalisib
Hematopoietic stem cell transplantation	Allogeneic stem cell transplantation At least 6 mo Longer duration may be required with chronic GVHD or ongoing immunosuppressive therapy Autologous stem cell transplantation Consider for 3–6 mo
Solid tumors^a^	Corticosteroids at prednisone-equivalent doses >20 mg/d for >4 wk Brain irradiation + temozolomide
Solid organ transplantation	Heart, liver, kidney, pancreas recipients For 6–12 mo Lung and small bowel recipients Lifelong
Autoimmune/inflammatory diseases^b^	Corticosteroids at prednisone-equivalent doses >20 mg/d for >4 wk Monotherapy (debated) Plus 1 of the following: additional immunosuppressive agents, steroid-sparing antirheumatic drugs, underlying immunosuppressive condition
Primary immune deficiency	Idiopathic CD4 lymphocytopenia Severe combined immunodeficiency X-linked hyper-IgM syndrome

Indications and practices are based on existing guidelines and evidence-supported practices [17–24]; inspired and adapted from Halani et al [25].

Abbreviations: GVHD, graft-vs-host disease; PJP, *Pneumocystis jirovecii* pneumonia.

^a^Considerations for PJP prophylaxis should also be given to patients treated with nucleoside analogs and those at higher risk from their chemotherapy regimen.

^b^There is no consensus guidelines for this patient population; indications described are accepted clinical practices [22–24]. This population includes others undergoing corticosteroid therapy.

Six patients (4.7%) developed PJP pneumonia while undergoing PJP prophylaxis at the time of diagnosis. All had an underlying hematologic disease, and only 2 were treated by steroids. Three received a combination of immunosuppressive agents, and 1 patient was treated by a calcineurin inhibitor. Interestingly, 3 patients (50%) received aerosolized pentamidine for their PJP prophylaxis. Knowing that aerosolized pentamidine is less effective than other regimens [26–28], this may have contributed to the development of PJP in those patients.

The effectiveness of PJP prophylaxis has otherwise been well established. Notably, in a 2014 Cochrane systematic review, Stern et al [7] looked at its effectiveness among non-HIV immunocompromised cases. It included 1412 patients with acute leukemia and solid organ transplantation from 13 trials. The authors found that prophylaxis with TMP-SMX reduced the occurrence of PJP by 85% without any increase in adverse events. Consequently, the number of patients who needed to be treated to prevent 1 episode of PJP was 19, while PJP infection occurred at a rate of 6% with no prophylaxis. Considering our results and given the low risks and good tolerance of PJP prophylaxis [7], we believe that such therapy is warranted in any individual receiving corticosteroids for >28 days at a daily prednisone-equivalent dose >20 mg, regardless of the underlying condition and the coadministration of other immunosuppressive therapies. Whether this recommendation should be extended to patients taking corticosteroids for >14 days and daily prednisone-equivalent doses superior to 15 mg represents an interesting future research question. Moreover, based on our findings, the prescription of a PJP prophylaxis should be considered in individuals receiving either multiple immunosuppressive agents or immunosuppressive agents in addition to corticosteroids, even at a prednisone-equivalent dose inferior to 20 mg/d. We strongly advocate for newer and more thorough consensus guidelines on the matter.

Our study has several limitations. First, its retrospective nature might lower the precision of the collected data. Second, all the recruited patients were diagnosed and treated in 1 of the 6 tertiary hospital centers of the Québec City region, which may have induced a selection and referral bias. Finally, patients with PJP diagnosed by a quantitative PCR assay were included. While we acknowledge that this comes with a theoretical risk of including asymptomatic carriers of *P jirovecii*, we believe that we have limited the risk by discussing every single case among us and ensuring careful review of clinical presentation, risk factors, and plausible alternate diagnoses.

Nonetheless, this study provides a well-needed update on the epidemiology of the *P jirovecii* pneumonia population. Our cohort is one of the largest reported in the literature focusing exclusively on HIV-negative cases. It supports the need for renewed interest in the prevention of PJP and its potentially deadly outcomes. Furthermore, it raises the question of whether most of those PJP cases could have been prevented with stricter adherence and broadening of current recommendations for prophylaxis.

## CONCLUSION

In summary, this study demonstrates the importance and significance of *P jirovecii* pneumonia in non-HIV immunocompromised cases. The use of corticosteroids—in monotherapy or in coadministration with other immunosuppressive agents—remains the principal risk factor. Corticosteroid doses >15 mg/d for >14 days are sufficient to cause PJP. Current prophylaxis guidelines and accepted practices are insufficient to adequately prevent a majority of infections and need to be broadened and updated. Further research and trials on the subject are needed.

## Supplementary Material

ofad639_Supplementary_Data

## Supplementary Data

Supplementary materials are available at *Open Forum Infectious Diseases* online. Consisting of data provided by the authors to benefit the reader, the posted materials are not copyedited and are the sole responsibility of the authors, so questions or comments should be addressed to the corresponding author.

## Notes

***Patient consent statement.*** The design of this work was approved by the institutional research ethics boards of the Institut Universitaire de Cardiologie et de Pneumologie de Québec and the Centre Hospitalier Universitaire de Québec.
