# Supplementary material for: Epidemiology, Risk Factors, and Prophylaxis Use for Pneumocystis jirovecii Pneumonia in the Non-HIV Population: A Retrospective Study in Québec, Canada
Source: Open Forum Infect Dis. 2023 Dec 18;11(1):ofad639. doi: 10.1093/ofid/ofad639 (PMC10810061; doi:10.1093/ofid/ofad639)
Supplement: ofad639_Supplementary_Data [file ofad639_supplementary_data.zip › Supplemental Table 2.docx]

**Supplemental Table 2 – Characteristics of patients who developed PJP infection despite receiving a prophylaxis.**

| Gender | Age | Underlying condition(s) | Corticosteroid use | Other immunosuppressants | Prophylaxis used |
| --- | --- | --- | --- | --- | --- |
| Male | 24 | Allogeneic hematopoietic stem cell transplantation (previous leukemia) | None | Cyclosporine | TMP-SMX 800 mg-600 mg orally 3 times per week |
| Male | 66 | Leukemia | None | Rituximab, fludarabine, cyclophosphamide | Aerosolized pentamidine 300 mg every 4 weeks |
| Male | 67 | Lymphoma | None | Bendamustine | Atovaquone 1500 mg orally daily |
| Female | 75 | Lymphoma, pemphigus vulgaris | Prednisone (15 to 50 mg daily for more than 160 days) | Fludarabine, cyclophosphamide, rituximab, cyclosporine | Aerosolized pentamidine 300 mg every 4 weeks |
| Female | 34 | Allogeneic hematopoietic stem cell transplantation (previous lymphoma) | None | Tacrolimus | Aerosolized pentamidine 300 mg every 4 weeks |
| Female | 64 | Hemophagocytic lymphohistiocytosis, vasculitis | Dexamethasone (20 mg daily for 28 days) | Etoposide, cyclosporine, methotrexate | TMP-SMX 800 mg-600 mg orally 3 times per week |
