# Supplementary material for: Epidemiology, Risk Factors, and Prophylaxis Use for Pneumocystis jirovecii Pneumonia in the Non-HIV Population: A Retrospective Study in Québec, Canada
Source: Open Forum Infect Dis. 2023 Dec 18;11(1):ofad639. doi: 10.1093/ofid/ofad639 (PMC10810061; doi:10.1093/ofid/ofad639)
Supplement: ofad639_Supplementary_Data [file ofad639_supplementary_data.zip › Supplemental Table 3.docx]

**Supplemental Table 3 – Characteristics of patients who developed PJP on immunosuppressants alone, without co-administration of corticosteroids.**

| Gender | Age | Underlying condition(s) | Immunosuppressants | PJP prophylaxis used |
| --- | --- | --- | --- | --- |
| Female | 81 | Rheumatoid arthritis | Methotrexate | None |
| Male | 67 | Heart transplant | Tacrolimus | None |
| Female | 72 | Psoriatic arthritis | Methotrexate + secukinumab | None |
| Female | 76 | Psoriatic arthritis | Methotrexate | None |
| Male | 24 | Allogeneic hematopoietic stem cell transplantation (previous leukemia) | Cyclosporine | TMP-SMX 800 mg-600 mg orally 3 times per week |
| Female | 59 | Rheumatoid arthritis | Methotrexate + tocilizumab + hydroxychloroquine | None |
| Male | 80 | Rheumatoid arthritis | Methotrexate | None |
| Male | 66 | Leukemia | Rituximab + fludarabine + cyclophosphamide | Aerosolized pentamidine 300 mg every 4 weeks |
| Male | 67 | Lymphoma | Bendamustine | Atovaquone 1500 mg orally daily |
| Female | 34 | Allogeneic hematopoietic stem cell transplantation (previous lymphoma) | Tacrolimus | Aerosolized pentamidine 300 mg every 4 weeks |
| Female | 54 | Lymphoma and myelodysplastic syndrome | Fludarabine + cyclophosphamide + rituximab | None |
| Female | 67 | Breast cancer | Everolimus + exemestane | None |
