# Supplementary material for: Epidemiology, Risk Factors, and Prophylaxis Use for Pneumocystis jirovecii Pneumonia in the Non-HIV Population: A Retrospective Study in Québec, Canada
Source: Open Forum Infect Dis. 2023 Dec 18;11(1):ofad639. doi: 10.1093/ofid/ofad639 (PMC10810061; doi:10.1093/ofid/ofad639)
Supplement: ofad639_Supplementary_Data [file ofad639_supplementary_data.zip › Supplemental Table 1.docx]

**Supplemental Table 1 – Characteristics of patients who developed PJP despite no recent immunosuppressant or corticosteroid use.**

| Gender | Age | Underlying condition(s) |
| --- | --- | --- |
| Male | 73 | Chronic myelomonocytic leukemia |
| Female | 74 | None identified |
| Female | 69 | Unclassified interstitial lung disease, remote gastric bypass surgery |
| Female | 71 | Scleroderma with associated interstitial lung disease |
| Male | 76 | Chronic lymphocytic leukemia (lymphocyte count 152 x 10^9^/L at time of PJP diagnosis) |
| Male | 88 | None identified |
| Male | 55 | Prior allogeneic hematopoietic stem cell transplantation |
| Male | 66 | Advanced lung cancer |
| Male | 74 | Lymphoma |
